# Post-translational regulation of rice MADS29 function: homodimerization or binary interactions with other seed-expressed MADS proteins modulate its translocation into the nucleus

**DOI:** 10.1093/jxb/eru296

**Published:** 2014-08-05

**Authors:** Saraswati Nayar, Meenu Kapoor, Sanjay Kapoor

**Affiliations:** ^1^Interdisciplinary Centre for Plant Genomics and Department of Plant Molecular Biology, University of Delhi South Campus, Benito Juarez Road, New Delhi 110021, India; ^2^University School of Biotechnology, Guru Gobind Singh Indraprastha University, Sector 16C, Dwarka, New Delhi 110078, India

**Keywords:** MADS box, nuclear localization, protein–protein interaction, rice, seed, transcription factor.

## Abstract

OsMADS29 is a seed-specific MADS-box transcription factor that affects embryo development and grain filling by maintaining hormone homeostasis and degradation of cells in the nucellus and nucellar projection. Although it has a bipartite nuclear localization signal (NLS) sequence, the transiently expressed OsMADS29 monomer does not localize specifically in the nucleus. Dimerization of the monomers alters the intracellular localization fate of the resulting OsMADS29 homodimer, which then translocates into the nucleus. By generating domain-specific deletions/mutations, we show that two conserved amino acids (lysine^23^ and arginine^24^) in the NLS are important for nuclear localization of the OsMADS29 homodimer. Furthermore, the analyses involving interaction of OsMADS29 with 30 seed-expressed rice MADS proteins revealed 19 more MADS-box proteins, including five E-class proteins, which interacted with OsMADS29. Eleven of these complexes were observed to be localized in the nucleus. Deletion analysis revealed that the KC region (K-box and C-terminal domain) plays a pivotal role in homodimerization. These data suggest that the biological function of *OsMADS29* may not only be regulated at the level of transcription and translation as reported earlier, but also at the post-translational level by way of the interaction between OsMADS29 monomers, and between OsMADS29 and other MADS-box proteins.

## Summary

OsMADS29, a seed-specific transcription factor that affects grain filling and embryo development by regulating hormone homeostasis, requires homo- or heterodimerization with eleven other MADS proteins for its localization into the nucleus.

## Introduction

MADS box proteins are homeotic transcription factors (TFs) that have been implicated in reproductive development in vascular plants (Kater *et al.*, 2006). Among these TFs, A, B, C, D, and E classes of genes have been extensively investigated with regards to their combinatorial effect on the control of flower development (Kater *et al.*, 2006, Zhang *et al.*, 2013). On the basis of structure and phylogeny, MADS box proteins have been divided into two major classes, Type I and Type II. The Type I class has been further subdivided into Mα, Mβ, and Mγ types, whereas the Type II comprises MIKC^C^ and MIKC* subclasses (Gramzow and Theissen, 2010). The MIKC is an acronym for the four conserved domains that these proteins are made up of, namely, MADS box, intervening region, K box, and C-terminal region (Becker and Theißen, 2003).

Like most proteins involved in regulatory functions, MADS proteins also undergo homo or hetero types of binary, as well as, higher order protein–protein interactions, which have been shown to be instrumental and essential for the determination of floral organ identity and precise regulation of specific target gene sets (Pawson and Nash, 2003; Tonaco *et al.*, 2006; Immink *et al.*, 2010). Physical interactions between A, B, C, and D class MADS-domain proteins have been shown to be mediated by the members of E class (SEPALLATA) proteins, which act as ‘bridging molecules’ to bring the interacting partners together (Immink *et al.*, 2009). Both, *in vitro* and *in vivo* methodologies have been used to study interactions between MADS proteins and their interacting partners. Where *in vitro* methodologies provide unambiguous evidence relating to structure and dynamics of the interaction, the *in vivo* methods like BiFC and FRET provide another dimension to the function of the complex by revealing their intracellular localization (Piehler, 2005; Howell *et al.*, 2006; Miernyk and Thelen, 2008). There is a general consensus that the K-domain (comprising three amphipathic α-helices, K1, K2, and K3) plays an important role in protein–protein interactions (Yang and Jack, 2004). However, the precise ‘K’ subdomain that takes part in an interaction seems to be dependent on the interacting partners. For example, the K2 subdomain, containing five conserved leucine residues, was found to be essential for the interaction between OsMADS6 and OsMADS14, whereas the K3 and the partial C domains in OsMADS6 had stabilizing effects on this interaction (Moon *et al.*, 1999). However, in case of two interactions involving APETALA1 (AP1) and SEPALLATA3 (SEP3) with a non-MADS transcriptional repressor, SEUSS, in *Arabidopsis,* the C-terminal domain instead of the K domain was found to be involved (Sridhar *et al.*, 2006). These data suggest that both K and C domains could be involved in protein–protein interactions. It is logical to think that there could be more than one protein interaction domains in MADS proteins, because in a ternary complex each polypeptide would have to interact with more than one interacting partner. Recently, using an integrated computational and experimental approach, short sequence motifs in the ‘I’ as well as the ‘K’ domains have been proposed to be involved in dimer interaction between two *Arabidopsis* SUPPRESSOR OF OVEREXPRESSION OF CO 1 (SOC1) polypeptides (van Dijk *et al.*, 2010).

As transcription factors, MADS-domain proteins are assumed to localize in the nucleus. Most MADS proteins have also been found with putative conserved bipartite nuclear localization signals (NLSs) as part of the MADS domain (Gramzow and Theissen, 2010). Moreover, nuclear localization of a number of MADS domain protein representatives from *Arabidopsis,* rice, soybean, and petunia have been validated experimentally (Immink *et al.*, 2002; Gramzow and Theissen, 2010). However, in the case of MADS proteins, e.g. FBP2 and FBP11, which localize to the nucleus as a dimer, functional NLSs in both interacting partners have been found to be necessary, because when either of the NLSs is compromised, the complex loses its capability to localize specifically to the nucleus (Immink *et al.*, 2002). These observations suggest that two different pathways could exist for MADS proteins to localize in the nucleus. Although some MADS proteins could enter the nucleus uninhibited in the monomeric form, others might need to dimerize with either MADS or non-MADS partners for their entry into the nucleus. And this requirement of protein–protein interaction for nuclear transport could serve as an added level of control, leading to enhanced functional specificity.

*OsMADS29* (from here on referred to as *M29*) is a type II MIKC^C^ B-sister class gene that has been implicated in embryo development and grain filling by maintaining hormone homeostasis and degradation of cells in the nucellus and nucellar projection (Yang *et al.*, 2012; Yin and Xue, 2012; Nayar *et al.*, 2013). Analyses involving temporal and spatial accumulation of mRNA and protein have shown that the expression of *M29* is tightly regulated at both transcription and translational levels (Nayar *et al.*, 2013). Whole genome transcriptome analyses of *M29* overexpression and knockdown lines indicated that a number of genes associated with pathways involving hormone biosynthesis/signalling, starch biosynthesis, plastid biogenesis, etc. are under its direct or indirect control (Nayar *et al.*, 2013). In the present study, we show that M29 monomers associate to form a homodimer and also interact with at least 19 other seed-expressed MADS domain proteins. Homo- and heterodimer complexes formed as a result of interaction between M29 monomers or between M29 monomers and eleven other MADS proteins localize specifically in the nucleus. Conversely, M29 monomers also form complexes with eight other MADS proteins, which localize mainly in the cytoplasm. We further demonstrate that the NLS sequences in both the monomers forming the homodimer are essential for effective localization of M29 to the nucleus. It is possible that by way of these interactions and specific regulatory controls on its expression, M29 is able to accomplish its function during seed development.

## Material and methods

### Construction of plasmid vectors for subcellular localization, interaction analysis, and domain analysis

The *OsMADS29* CDS was amplified from KOME clone AK109522 using gene-specific primers with additional *Apa*I and *Xma*I restriction sequences in the forward and reverse primers, respectively, using Pfu DNA polymerase. After restriction digestion with *Apa*I and *Xma*I and gel purification, the CDS amplicon was cloned in pGFPcs_1/pUC18 vector (Jiang *et al.*, 2001). For cloning in BiFC Gateway^TM^ vectors, pDH51-GW-YFPc (N9843) and pDH51-GW-YFPn (N9842) (Zhong *et al.*, 2008), complete CDSes of 30 MIKC MADS genes (without stop codon) were amplified using seed cDNA or KOME clones by using Phusion High Fidelity Taq polymerase (Finnzymes) with additional ‘CACC’ at the 5′ end in the forward primer for cloning in the pENTR/D-TOPO vector (Invitrogen Inc. USA) as per the manufacturer’s protocol. These inserts were then transferred to BiFC destination vectors using Gateway™ LR clonase™ enzyme mix. Primers used for these amplification reactions are listed in supplementary Table S1. The M29 variants; M29^∆MADS^ (deletion of 2–70 residues of the MADS domain), M29^NLS-MUT^ (complete cds with K23A and R24A mutations), M29^∆KC^ (deletion of 79–260 residues comprising K-box and C-terminal domain) and M29^CLR-MUT^ (complete cds with L144A, L148A, L151A, L158A, and L165A mutations), were amplified from the full-length *OsMADS29* KOME clone (AK109522) by using specific primers (supplementary Table S1) and Phusion^TM^ high-fidelity Taq polymerase (Finnzymes) with additional ‘CACC’ at the 5′ in the forward primer for cloning in pENTR/D-TOPO vector (Invitrogen Inc. USA) as per the manufacturer’s protocol.

### Intracellular localization

Particle bombardment was carried out using Biolistic PDS-1000/He particle delivery system (Bio-Rad, USA) according to the protocol described earlier (Lee *et al.*, 2008b) with minor modifications. For each construct 2.5 µg of DNA was used to coat 1.5mg (0.5mg per shot) 1 µm gold particles and the following shooting parameters were used: 27mm Hg vacuum, 1100 psi helium pressure, and target distance of 9cm. The plates were incubated for 10−12h at 28 °C, in the dark. The onion peels were observed for GFP and YFP expression under a fluorescence microscope with I3 filter cube (excitation filter, BP 450−490; dichromatic filter, 510 and suppression filter, LP515, DM 5000B, Leica Gmbh, Germany). The bright field as well as fluorescence images were taken separately and overlaid by using Adobe^®^ Photoshop CS5.

### Yeast two-hybrid analysis

From pENTR/D-TOPO vector the *M29* CDS was cloned into bait vector pDEST-GBKT7 and all BiFC-positive interactors of M29 were cloned from pENTR/D-TOPO vector into prey vector pDEST-GADT7 using Gateway™ LR clonase™ enzyme mix (Invitrogen). The bait vector was used to transform Y187 yeast cells and prey vectors were immobilized in the Y2H-Gold strain of yeast and selected on SD medium lacking Trp and Leu, respectively, as per the protocol supplied by the manufacturer. Further, overnight cultures for mating were grown from single colonies of bait and prey yeast transformants in 500 µl of 2×YPDA at 30 °C and 200rpm. Droplets of 20 µl were placed on the selection media (SD/-Leu-Trp, SD/-His-Leu-Trp, SD/-His-Leu-Ade-Trp and SD/-His-Leu-Ade-Trp supplemented with X-α-gal) and the colonies were allowed to grow for 3–8 days at 30 °C.

The yeast two-hybrid α-Gal quantitative assay was performed to quantitate the strength of interaction between the interacting proteins. The catalytic activity of α-galactosidase was assayed by measuring the rate of hydrolysis of the chromogenic substrate p-nitrophenyl- α-d-galactoside (PNP- α-Gal) to p-nitrophenol according to the manufacturer’s protocol (Clontech). Fresh colonies were inoculated in SD/-Leu-Trp liquid culture and incubated at 250rpm and 30 °C, overnight. Cells with OD_600_ 0.5–1 were pelleted and the supernatant was incubated with the assay buffer containing sodium acetate (pH 4.5) and PNP- α-Gal for 1h at 30 °C. The reaction was stopped with 1× stop buffer containing sodium carbonate and OD was recorded at 410nm. The α-galactosidase units were calculated based on manufacturer’s protocol (Clontech).

## Results

### Nuclear localization of OsMADS29: monomeric vs homodimeric form

**Fig. 1.**
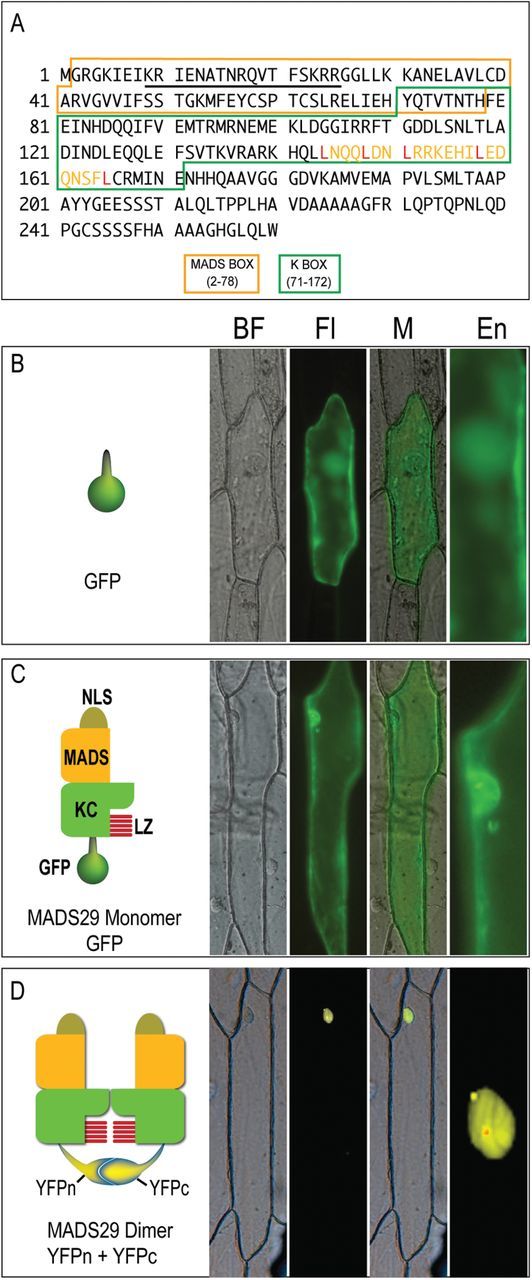
Intracellular localization of M29 monomer and homodimer. (A) M29 polypeptide sequence showing the position of conserved domains and motifs. The MADS, and K-box domains are marked by different coloured boxes. The unmarked region represents the C-terminal domain. The NLS sequence is underlined and the leucine-zipper-like motif in the K-box has been shown by yellow font with five conserved leucine residues marked in red. (B–D) The left subpanels in panels B, C, and D contain graphical representations of the M29:reporter constructs used in this experiment, while the actual expression of these chimeric proteins in onion epidermal cells is shown in the respective right subpanels. (B) GFP (C) M29:GFP (D) M29:YFPc+M29:YFPc. BF, bright field; Fl, fluorescence image; M, Merged image; En, enlarged view of the nuclear region.

Based on the Conserved Domain Database (CDD) analysis, *M29* codes for a 260-amino acid (~29kDa) polypeptide, and consist of a MADS Type II/ MEF2-like motif (2–78 amino acids), K box (71–171 amino acids), and C-terminal domain (172–260 amino acids; http://www.ncbi.nlm.nih.gov/Structure/cdd/wrpsb.cgi; Marchler-Bauer *et al.*, 2011). The MADS box domain also contains a bipartite nuclear localization signal (17 amino acids) as predicted by “WoLF PSORT” (http://wolfpsort.org; Horton *et al.*, 2007) and the K-box region harbours a leucine-zipper-like motif (144–166 amino acids, with conserved leucines at positions 144, 148, 151, 158, and 165; Fig. 1A; Lim *et al.*, 2000).

For being a member of the MADS-box class of TFs and possessing a bipartite nuclear localization signal, M29 was expected to localize in the nucleus. Transient expression of GFP-tagged M29 fusion protein was therefore analysed in onion epidermal cells. Similar to GFP, M29:GFP fusion protein was observed to be uniformly distributed in both the nucleus and the cytoplasm (Fig. 1B, C). This suggested that either (i) M29 was not a nuclear localized protein or (ii) it needed to interact with other proteins to get translocated into the nucleus.

As a number of MIKC^c^ MADS proteins are known to interact with either themselves or other proteins to form dimers or higher order complexes (de Folter *et al.*, 2005; Kater *et al.*, 2006; Arora *et al.*, 2007), we first examined the possibility of MADS29 being involved in homodimer formation and if this self-association had any bearing on its intracellular localization. A BiFC vector pair with M29 fused either to N- or C-terminal halves of YFP (M29:YFPn and M29:YFPc) was prepared and used to co-transfect onion peel cells. Fluorescence emitted by YFP clearly indicated specific interaction between the two M29 monomers. The homodimer thus formed was observed to be specifically localized in the nucleus (Fig. 1D). These results, therefore, demonstrate that dimerization of M29 monomers plays an important role in retaining M29 in the nucleus. Whether M29 dimerizes to gain entry into the nucleus or it translocates into the nucleus in the monomeric form and then interacts with other M29 molecule is presently not known.

### Interactions of M29 with other seed-expressed MIKC MADS-domain proteins

**Fig. 2.**
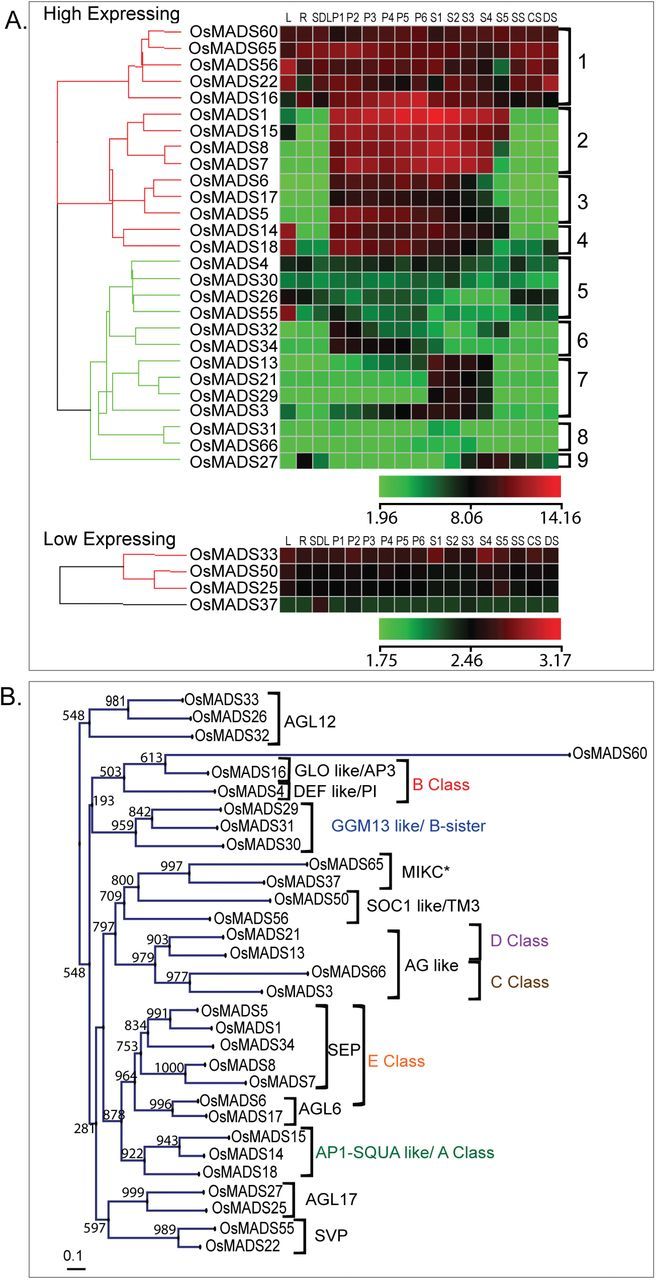
Transcript accumulation patterns and phylogenetic relationships of seed-expressed rice MADS-box genes. (A) Hierarchical cluster display of expression profiles for seed-expressed rice MADS-box genes chosen for protein–protein interaction analysis with M29. L, leaf; R, root; SDL, seedling; P1 to P6, panicle stages; S1 to S5, seed stages; CS, SS, DS: cold, salinity and drought stress stages. Colour bars below each sub-panel represent the range of expression values in log_2_. (B) Phylogenetic relationship of the genes shown in panel A and their distribution in various MIKC sub-clades based on Arora *et al.* (2007).

As MADS domain proteins are known to interact with multiple proteins and have been proposed to bind to target regulatory sequences as quartets (Theißen and Saedler, 2001; Gramzow and Theissen, 2010), we identified 30 MADS genes as putative M29 interactors as they were found to express in at least one of the five (S1–S5) stages of seed development (Fig. 2A). Selection of the expression window was based on our earlier findings relating to the transcript and protein accumulation patterns of *M29* (Nayar *et al.*, 2013). The expression patterns of *OsMADS13* and *OsMADS21* overlapped significantly with that of *M29* in being predominantly seed specific, but most other genes were expressed in stages/tissues other than those related to seed development as well (Fig. 2A). The phylogenetic affiliations of the genes selected for this analysis to the floral homeotic and MIKC^C^ and MIKC* sub-clade of rice MADS genes is shown as Fig. 2B to serve as a reference point for subsequent discussion. (Arora *et al.*, 2007)).

**Fig. 3.**
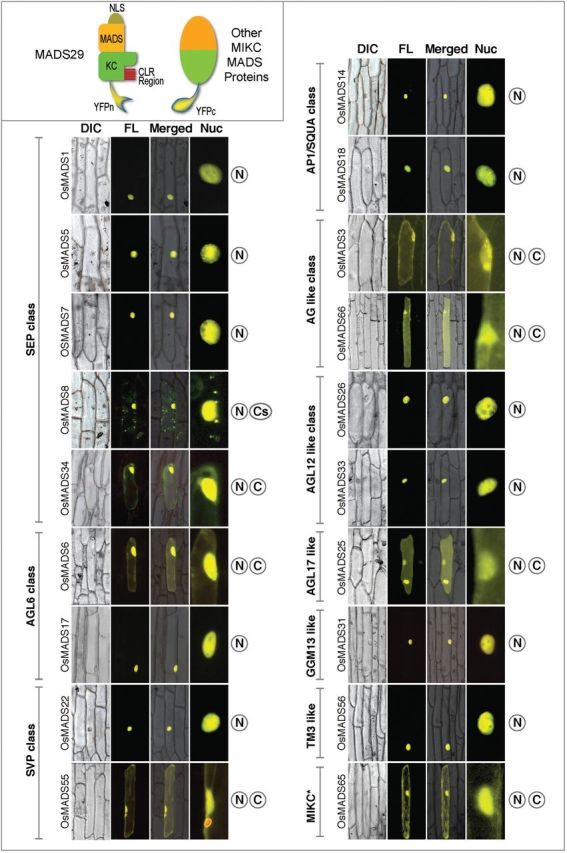
Identification and intracellular localization of 19 seed-expressed M29-interacting MADS domain proteins. The phylogenetic affiliation to MIKC subclades is mentioned on the left, whereas the domains of cellular localization (N, nuclear; C, cytoplasmic; Cs, cytoplasmic speckles) are mentioned on the right side of each panel. In this analysis, M29 was tagged with YPFn and putative interacting partners were tagged with YFPc. DIC, bright field DIC; Fl, fluorescence; M, merged, Nuc; enlarged image of the nuclear region.

Out of the 30 selected genes, coding sequences of 24 genes were sub-cloned from the respective KOME clones, whereas for the other six, full-length cDNAs were amplified from the RNA prepared from rice (IR64) panicle tissue and cloned in BiFC and Y2H vectors. The BiFC partner pairs, consisting of M29:YFPn and each one of the putative interactors linked to YFPc, were used to co-transfect onion peel cells to not only identify the interacting partners but to also analyse the effects of these interactions on the ability of the resultant dimers to translocate into the nucleus. These analyses revealed that, interactions between two M29 monomers, the M29 monomers interacted with at least 19 other seed expressing MADS-domain proteins (Fig. 3). These included all five *SEP* genes, *OsMADS1, 5, 7, 8*, and *34*; two *AGL6* genes, *OsMADS6* and *17*; two *SVP* genes, *OsMADS22* and *55*; two *AP1/SQUA* genes, *OsMADS18* and *14*; two AG-like genes, *OsMADS3* and *66*; two *AGL12*-like genes, *OsMADS26* and *33*; *AGL17-*like gene, *OsMADS25*; *GGM13/B-sister* like gene, *OsMADS31*; *TM3-*like gene, *OsMADS56*; and an MIKC* gene, *OsMADS65.*

Three different patterns of cellular localization were observed for the M29 dimers. Eleven of these dimers, where M29 interacted with OsMADS1, 5, 7, 14, 17, 18, 22, 26, 31, 33, and 56 were observed to be localized specifically in the nucleus, whereas the fluorescence signal emanating from seven M29 dimers with OsMADS3, 6, 25, 34, 55, 65, and 66 was evenly distributed in the nuclear and the cytoplasmic compartments. Distinctively, the M29::OsMADS8 dimers were found to be localized primarily in the nucleus; however, a small proportion also seemed to be aggregated as cytoplasmic speckles (Fig. 3). Interaction of M29 with M29, OsMADS3, 8, and 14 are also predicted by the “Predicted Rice Interactome Network” tool (PRIN, http://bis.zju.edu.cn/prin/; Gu *et al.*, 2011).

### Validation of M29 interactions by yeast-two-hybrid

**Fig. 4.**
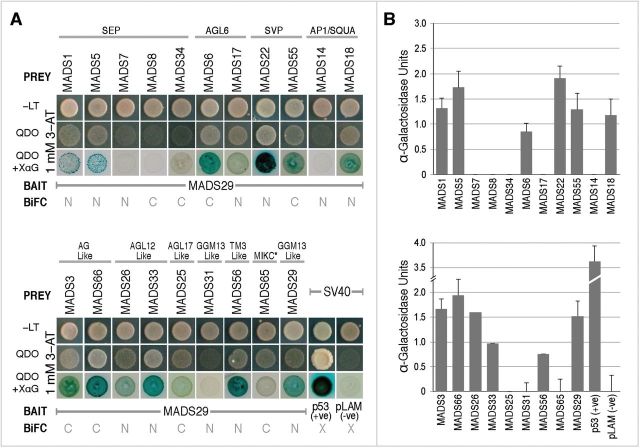
Yeast two-hybrid based validation of positive interactions obtained from BiFC analysis. (A) The Y2H assays where M29 is cloned in bait vector and the cds of all other positive interactors are cloned in the prey vector. Binary interactions between bait and prey proteins were observed by mating and subsequent growth on –LT, quadruple drop out (QDO) and QDO+X-alpha-Gal (XαG). (B) α-Galactosidase quantitative assay for monitoring catalytic activity of the endogenous *MELI* reporter gene in liquid media, which confirms the positive interaction between bait and prey proteins and successful activation of *Gal4* responsive promoter. The αGalactosidase activity is indicative of relative strengths of the interactions.

To confirm M29 interactions by Y2H assays, full-length coding sequences of positive interactors, identified by BiFC analysis, were cloned in yeast-two-hybrid prey vector, pDEST-GADT7, downstream of the GAL4 activation domain, whereas that of M29 was fused downstream of the GAL4 DNA-binding domain in the bait vector pDEST-GBKT7. Interactions between M29 and the two SEP proteins, OsMADS1 and 5; the AGL6-like proteins, OsMADS6 and 17; the SVP-like proteins, OsMADS22 and 55; the AG-like proteins, OsMADS3 and 66; the AGL12-like proteins, OsMADS26 and 33; the AGL17-like protein, OsMADS25; the TM3-like protein, OsMADS56 and another M29 subunit yielded positive results with viable colonies on quadruple drop out (QDO) medium (Fig. 4A). The interactions between the M29 monomer and its interactors were further confirmed with the development of viable blue coloured colonies on QDO+X-α-Gal (XαG) medium. The strength of interactions between the proteins was visually interpreted from the intensity of the blue colour, which was found to be the strongest in case of OsMADS22 and the weakest for OsMADS17 and 25.

To quantitate the strength of the observed interactions, α-galactosidase assays were also performed (Fig. 4B). The complexes of M29 with OsMADS1, 3, 5, 18, 22, 26, 29, 55, and 66 were found to be relatively stronger than those with OsMADS 6, 31, 33, 56, and 65. The α-galactosidase activity for M29 complexes with OsMADS17 and 25 could not be determined, probably because these were beyond the sensitivity limits of the assay. Also, we did not observe any apparent correlation between the strength of interaction and the subcellular localization patterns of the M29 complexes in the nuclear and/or cytoplasmic compartments of the cell.

**Fig. 5.**
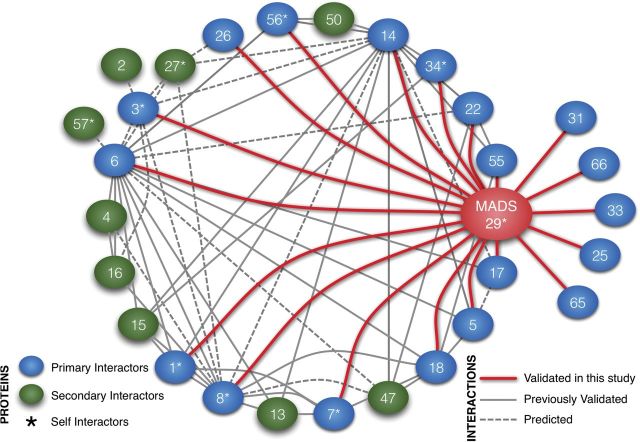
A graphical representation of validated as well as predicted interactions amongst seed-expressed MADS-box genes. The MADS proteins are shown as blue (primary M29 interactors validated in this study) and green (secondary interactors known from PRIN database; (Gu *et al.*, 2011; Ho *et al.*, 2012)) ellipses. The keys to connectors and nodes are shown as two bottom panels; self-interacting proteins are marked with asterisks (*).

On the basis of the data obtained in this study and that already available in the literature regarding protein–protein interactions of seed-expressed rice MADS box genes (Gu *et al.*, 2011; Ho *et al.*, 2012), a snapshot of the current state of protein–protein interaction network is summarized in Fig. 5. At present, M29 seems to have the highest (20) number of interacting partners, followed by MADS6, MADS14, and MADS8, with 11, 11, and 9 validated interactions, respectively. The high number of M29 interactors is suggestive of the diverse roles it might play in different cell types and during different stages of seed development in rice.

### Delineation of M29 conserved domains for their roles in nuclear localization and homodimer formation

**Fig. 6.**
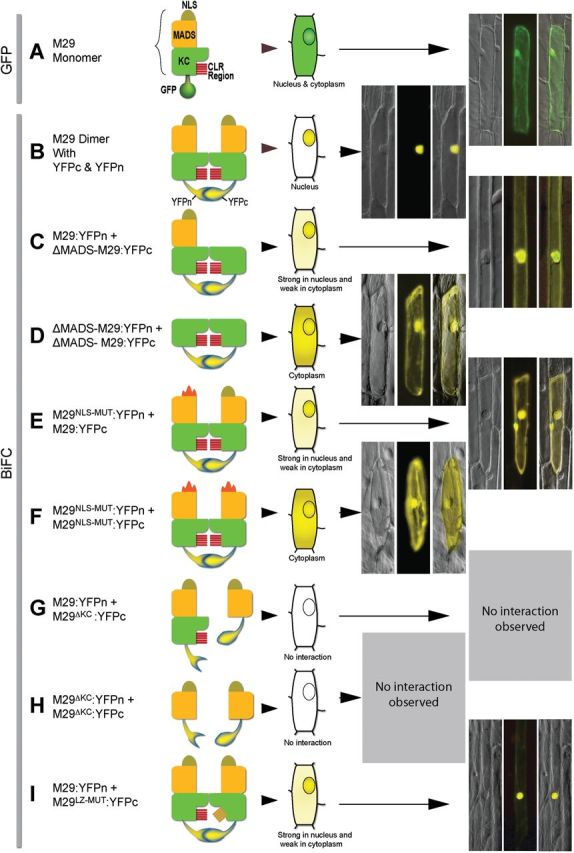
Delineation of MADS29 domains required for nuclear localization and homodimerization. In panels A–I, description of the interacting partners and their graphical representations are shown on the left, and the cartoons showing sub-cellular localization along with the actual fluorescence micrographs are shown on the right. Each fluoro-micrograph panel consists of the DIC (left) fluorescence (centre), and the overlaid (right) image sub-panels. The scheme of domain representation (MADS domain, NLS, the KC region, and the leucine zipper) for the M29 monomer is shown in panel A. The GFP (green fluorescent protein), YFPn (yellow fluorescent protein N-terminal half) and YFPc (yellow fluorescent protein C-terminal half; as marked in panels A and B) show the identity of fluorescent fusion proteins fused to the C-terminal ends of different M29 monomers. The mutated NLS and leucine zipper (LZ)-like domain are shown as altered graphics in panels E, F, and I.

To delineate the regulatory regions necessary for mediating the observed interactions between M29 monomers and between M29 and other proteins, deletion/site-directed mutation studies were carried out. Seven deletion or substitution variants of M29 were generated and their ability to influence nuclear localization and/or protein–protein interaction patterns were analysed using the BiFC approach (Table 1; Fig. 6). Deletion of the MADS domain from one or both of the interacting M29 monomers has detrimental effects on the ability of the dimer to remain localized in the nucleus (Fig. 6C, D). Unlike the concentrated YPF fluorescence observed in the nucleus, in the case of interaction of undeleted M29 monomers, the greenish-yellow YFP fluorescence was observed to spread out into the cytoplasm when the MADS domain from any of the M29 interacting monomers was deleted. The MADS domain possesses a 17 amino acid bipartite NLS towards its N-terminal end (8–25 amino acids). By using site-directed mutagenesis, the conserved lysine^23^ and arginine^24^ residues in the P2 region of the putative NLS (Lange *et al.*, 2007) of one or both the M29 monomers (linked to YFPn and YFPc) were replaced with the uncharged hydrophobic amino acid alanine (*see* Fig. 1A). Mutations in the NLS clearly disrupted the nuclear localization fate of the M29 homodimer (Fig. 6E, F). These data suggest that the NLS with lysine 23 and arginine 24 and not the rest of the MADS domain are critical for nuclear localization of the M29 dimer. The MADS domain and the NLS, however, do not interfere with ability of M29 to self-associate and probably interact with other MADS proteins as well. Furthermore, both the NLSs of the interacting M29 monomers function independently yet in a cooperative manner for translocating the homodimer into the nucleus.

**Table 1.** Details of M29 variants used in BiFC-based dimerization analysis

	M29 variant dimers	Variation details
1	M29:YFPn + M29^∆MADS^:YFPc	Deletion of 2–70 residues of MADS domain in one of the monomers
2	M29^∆MADS^:YFPn + M29^∆MADS^:YFPc	MADS region deleted in both the interacting monomers
3	M29^NLS-MUT^:YFPn + M29^NLS-MUT^:YFPc	Conversion of the lysine23 and arginine24 residues in the conserved NLS regions of both the M29 monomers to uncharged hydrophobic amino acid, alanine (K23A and R24A)
4	M29:YFPn + M29^NLS-MUT^:YFPc	Conversion of the lysine23 and arginine24 residues in the conserved NLS regions of one of the M29 monomers to uncharged hydrophobic amino acid, alanine (K23A and R24A)
5	M29:YFPn + M29^∆KC^:YFPc	Deletion of 79–260 residues comprising K-box and C-terminal domain from one of the monomers
6	M29^∆KC^:YFPn + M29^∆KC^:YFPc	The KC region deleted from both the monomers
7	M29:YFPn + M29^LZ-MUT^:YFPc	Complete cds with L144A, L148A, L151A, L158A and L165A mutations in one of the monomers (see Fig. 1A).

As the MADS region did not show any effect on homodimerization, the conserved KC region and the predicted leucine zipper-like domain were targeted for functional delineation (Fig. 6G–I). The deletion constructs were prepared, where both the conserved K box and the C-terminal domains were deleted (M29^∆KC^) in one or both the monomers, or the leucine zipper-like domain was mutated (M29^LZ-MUT^) in one of the interacting monomers. In the M29^LZ-MUT^ construct, as five of the hydrophilic leucines were mutated to hydrophobic alanines, we first checked for the changes, if any, in the 3D structure of the predicted protein by using the web-based I-TASSER protein structure prediction application (Roy *et al*., 2010; 2012). The predicted M29^LZ-MUT^ had minor structural alterations, such as absence of a beta sheet, increased compactness, negative coiling of the leucine zipper, and less protrusion of the NLS as compared with the protein having intact leucine zipper-like domain (Supplementary Fig. S1). The interaction studies using these constructs revealed that, (a) in the absence of the KC region, which also harbours the leucine zipper-like domain, no interaction between the M29 monomers was observed, indicating that this region is indispensable for dimerization (Fig. 6G, H); (b) mutation in the leucine zipper region did not have any apparent effect on M29 dimerization. There was a minor loss in the specificity of nuclear localization for the M29:YFPn + M29^LZ-MUT^:YFPc dimer (Fig. 6I). These data suggest that instead of having a direct role in homodimerization, the LZ-like domain may help in maintaining a 3D conformation that favours nuclear localization. Further work, however, would be required to know if these domains play similar roles during heterodimerization of M29 with other MADS protein as well.

## Discussion

A number of MADS proteins have been shown to localize in the nucleus in transient assays, which may imply that these TFs can make use of general components of the nuclear transport machinery to translocate into the nucleus and their activities may not be regulated at the step of nuclear translocation. On the other hand, there are proteins like FLORAL BINDING PROTEIN 11 (FBP11), FBP9, and UNSHAVEN (UNS) in *Petunia* and SUPPRESSOR OF OVEREXPRESSION OF CONSTANS (SOC1), APETALA3 (AP3), and PISTILLATA (PI) in *Arabidopsis*, which need to interact with other MADS proteins for their translocation into the nucleus (McGonigle *et al.*, 1996; Immink *et al.*, 2002; Ferrario *et al.*, 2004; Lee *et al.*, 2008a). On the basis of the nuclear localization capabilities of the interacting proteins, these interactions can be classified into two types. One, where both the interacting partners, by themselves, are incapable of translocating into the nucleus but their dimers localize in the nucleus, e.g. AP3-PI (McGonigle *et al.*, 1996) and UNS-FBP9 (Ferrario *et al.*, 2004), whereas, in the second type, one of the interacting partners is unable to localize in the nucleus, by itself, but the other is nuclear localized, even as a monomer. The dimers of the two, however, localize in the nucleus. For example, FBP11 monomer as well as its homodimer are retained in the cytoplasm; however, its dimers with two SEP-class proteins FBP2 and FBP5 localize specifically in the nucleus (Immink *et al.*, 2002). Similarly, the monomeric SOC1 is retained in the cytoplasm, whereas its dimer with AGL24 is translocated to the nucleus (Lee *et al.*, 2008a). Our results show that although M29 cannot translocate into the nucleus in the monomeric form, its homodimer localizes in the nucleus, unlike FBP11. This might suggest that M29 homodimerization causes a conformational change, which exposes the NLS and brings it into a biologically active, nuclear-translocating conformation (Lee *et al.*, 2008a). We have also shown that M29 gets into this nuclear-translocating conformation with eleven other seed-expressed MADS proteins. Besides these, we have found eight more interactions that do not result in nucleus-specific translocation of the resultant M29 heterodimers. It would be interesting to know how many of these interactions are biologically relevant and whether complexes that are retained in the cytoplasm are involved in some cytosol-specific functions, or their role is just to prevent M29 from entering into the nucleus and performing its role as a transcriptional regulator.

### Role of MADS29 conserved domains in homodimerization and nuclear localization

In the case of M29, deletion of the KC half of the protein inhibited homodimerization; however, mutations in the leucine zipper-like domain (Yang and Jack, 2004) did not have any apparent effect on dimerization. Rather, a limited effect of the mutated LZ-like domain was seen on the nuclear localization of the homodimer. The leucine zipper domain has otherwise been known to facilitate homodimerization of STEROL REGULATORY ELEMENT-BINDING PROTEIN 2 (SREBP-2) (Nagoshi and Yoneda, 2001) and AKAP-Lbc (Baisamy, 2005), as well as heterodimerization of Jun and Fos (Chida *et al.*, 1999), and OsMADS6 and OsMADS14 (Moon *et al.*, 1999). Therefore, although it has not been found to affect M29 homodimerization, the role of the LZ-like region in heterodimerization cannot be completely ruled out. Earlier works have also demonstrated the importance of K1 and K2 helices in the K-box regions during interaction between two B-class MADS proteins, AP3 and PI (Yang *et al.*, 2003). The C-terminal domain, on the other hand, has been shown to be involved in the interactions of two *Arabidopsis* MADS protein AP1 and SEP3 with transcription co-repressors LEUNIG (LUG) and SEUSS (SEU) (Sridhar *et al.*, 2006). Therefore, the involvement of the KC region in dimerization of two MADS proteins is not unprecedented. However, it would be interesting to analyse which of these specific domains (or sub-domains) are involved in homo- and or heterodimerization functions. And also, if there exists a bias in these domains for specific interacting partners, which in turn may affect cellular localization of the resultant dimers.

MADS29, as previously discussed, consists of the MADS domain, which harbours a bipartite NLS. Point mutations in just two hydrophobic amino acids (K23A and R24A) in the NLS were sufficient to prevent nucleus-specific localization of the M29 homodimer. Our data suggest that, at least in case of the M29 homodimer, two functional NLSs are required for the efficient nuclear localization of the dimer. A similar scenario was reported earlier for FBP2 as well, where a single mutation in the FBP2 NLS severely affected nuclear localization of the homodimer and its heterodimer with FBP11 (Immink *et al.*, 2002). These data suggest that both the NLS regions might act co-operatively during nuclear transport of even those MADS-dimer partners, which do not localize to the nucleus in monomeric form.

### MADS29 also interacts with other seed-expressed MADS proteins

MADS29 was found to interact with some of the proteins, which already have a defined role during various stages of rice development. It interacted with all five rice E-class SEP proteins. In *Arabidopsis*, SEP proteins have been suggested to act as catalysts for multimeric MADS complex formation, because, of the 106 multimeric complexes involving MADS proteins, more than half have at least one of the SEPs as an interacting partner (Immink *et al.*, 2009). As far as specific dimers are concerned, the three *Arabidopsis* SEP proteins SEP1, SEP2, and SEP3 interact with 17, 5, and 13 other MADS proteins, respectively. Therefore, interaction of M29 with 19 other seed-expressed MADS proteins is suggestive of the central role it might play in regulating various aspects of seed development.

In rice, the E-class genes have been shown to be involved mainly in meristem and floral development-related functions, such as, maintenance of meristem identity, transition of vegetative to floral meristem, floral-organ identity, floral determinacy, etc. (Kang *et al.*, 1997; Jeon *et al.*, 2000; Jia *et al.*, 2000; Prasad *et al.*, 2001; Agrawal *et al.*, 2005; Prasad *et al.*, 2005; Ohmori *et al.*, 2009; Kobayashi *et al.*, 2010; Cui *et al.*, 2010; Li *et al.*, 2010; Gao *et al.*, 2010; Duan *et al.*, 2012). Our results show that M29 interacts with all five E-class genes. And, as all the E-class genes express in seed tissues (Arora *et al.*, 2007), it is possible that the interactions between M29 and specific E-class protein(s) might be central to the assembly of quaternary complexes involved in the regulation of seed-specific functions.

Besides E-class proteins, M29 also forms binary complexes with two AGL6-like (OsMADS6, 17), two SVP/STMADS1-like (OsMADS22, 55), two AP1/SQUA (OsMADS14, 18), two AG-like (OsMADS3, 66), two AGL12-like (OsMADS26, 33), and one each of AGL17 (OsMADS25), GGM13 (OsMADS31), and TM3-like (OsMADS56) proteins, along with an MIKC* protein (MADS65). Of these 14 proteins, six (OsMADS25, 26, 31, 33, 65, and 66) have not yet been assigned any function during vegetative or reproductive development, whereas others have been shown to be involved in mostly flowering time control, (which also involves transition of SAM into inflorescence meristem), meristem development, and control of floral organ identity specification (Moon *et al.*, 1999; Lee *et al.*, 2008c; Ryu *et al.*, 2009; Li *et al.*, 2011; Lee *et al.*, 2012). *OsMADS6,* which is an AGL6-like gene (with E-function), is the only M29 interactor that has been implicated in endosperm development during grain-filling stage by targeting a starch biosynthesis gene coding for ADP–glucose pyrophosphorylase (Zhang *et al.*, 2010). As the functions of the specific complexes involving these proteins and M29 are not known during seed development, as one of the target functions, their involvement in the establishment of meristematic zones in the developing embryo, would be worth exploring.

In conclusion, our data have demonstrated that nuclear localization of OsMADS29 is dependent on the interaction between M29 monomers and 11 other seed expressed MADS proteins. Its interactions with eight other MADS proteins, however, does not result in specific translocation into the nucleus. Furthermore, we have shown that functional NLS sequences in both the OsMADS29 monomers are necessary for optimal nuclear translocation capability of the homodimer. We have earlier shown that the expression of OsMADS29 is regulated at the levels of both (i) transcription (as its transcript accumulation is specific to seeds and is triggered after fertilization by a not-yet characterized pathway), and (ii) translation (because the M29 protein can only be detected at least after 4 DAP, whereas, its transcripts are present from 1 DAP onwards) (Nayar *et al.*, 2013). The data presented here strongly point towards the possibility of M29 being regulated also at the post-translational level by way of its interactions with several seed-expressed MADS proteins, which may influence its function by regulating its entry into the nucleus and eventually regulation of its targets. These data not only open up a new chapter for investigating the role of a cereal-specific regulator of seed development, but might also reveal new secrets concerning the roles of SEPs and other MADS proteins during grain filling and embryo development in cereals.

## Supplementary data

Supplementary data are available at *JXB* online

Fig. S1. Representation of mutated motifs in MADS29 domains for studying their importance in homodimer localization and dimerization. (A) Mutation of lysine and arginine to alanine in the NLS. (B) Leucine to alanine mutation in leucine zipper present in IKC domain. (C) Comparison of native protein and mutated (Leucine zipper) protein modelled using the web-based I-TASSER prediction program.

Table S1. List of primers used for DNA amplification/sequencing.

Supplementary Data
