# Supplementary material for: Post-translational regulation of rice MADS29 function: homodimerization or binary interactions with other seed-expressed MADS proteins modulate its translocation into the nucleus
Source: J Exp Bot. 2014 Aug 5;65(18):5339–50. doi: 10.1093/jxb/eru296 (PMC4157715; doi:10.1093/jxb/eru296)
Supplement: Supplementary Data [file supp_65_18_5339__index.html]

Post-translational regulation of rice MADS29 function: homodimerization or binary interactions with other seed-expressed MADS proteins modulate its translocation into the nucleus — Post-translational regulation of rice MADS29 function: homodimerization or binary interactions with other seed-expressed MADS proteins modulate its translocation into the nucleus — Supplementary Data 

# Post-translational regulation of rice MADS29 function: homodimerization or binary interactions with other seed-expressed MADS proteins modulate its translocation into the nucleus

## Supplementary Data

Data files

**Files in this Data Supplement:**

- Supplementary Data - Supplementary Data
