# Supplementary material for: Post-translational regulation of rice MADS29 function: homodimerization or binary interactions with other seed-expressed MADS proteins modulate its translocation into the nucleus
Source: J Exp Bot. 2014 Aug 5;65(18):5339–50. doi: 10.1093/jxb/eru296 (PMC4157715; doi:10.1093/jxb/eru296)
Supplement: Supplementary Data [file supp_eru296_jexbot124214_file001.pdf]

A.

M G R G K I E I K R I E N A T N R Q V T F S K R R G G L L

ATG GGG CGC GGC AAG ATC GAG ATC *AAG AGG ATC GAG AAC GCG ACG AAC AGG CAG GTG ACA TTC TCG* ***AAG AGG*** *CGG* GGA GGG CTA CTG

ATG GGG CGC GGC AAG ATC GAG ATC AAG AGG ATC GAG AAC GCG ACG AAC AGG CAG GTG ACA TTC TCG **GC**G **GC**G CGG GGA GGG CTA CTG

B.

L N Q Q L D N L R R K E H I L E D Q N S F L

CTG AAC CAG CAG **CTG** GAC AAC **CTT** CGT CGC AAG GAG CAC ATC **CTG** GAA GAC CAG AAC AGC TTC **CTG**

GCT AAC CAG CAG **GCT** GAC AAC **GCT** CGT CGC AAG GAG CAC ATC **GCT** GAA GAC CAG AAC AGC TTC **GCT**

C.

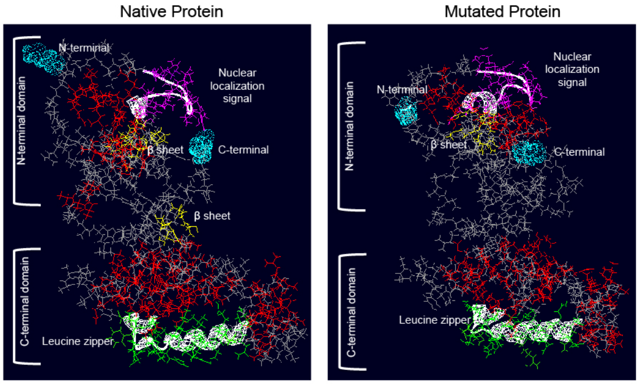

Figure S1. Representation of mutated motifs in MADS29 domains for studying their importance in homo dimer localization and dimerization (A) Mutation of lysine and arginine to alanine in the NLS (B) Leucine to alanine mutation in leucine zipper present in IKC domain (C) Comparison of native protein and mutated (Leucine zipper) protein modeled using web based I-TASSER prediction program.

Table S1: List of primers used for DNA amplification/sequencing

Gene ID/ Primer name	Forward Primer (5' to 3')	Reverse Primer (5' to 3')
M13 (TOPO) (for sequencing)	GTAAAACGACGGCCAG	CAGGAAACAGCTATGAC
M29ΔMADS	CACCATGTACCAGACCGTCACCAACACTCAT	
M29ΔKC		ATGAGTGTTGGTGACGGTCTGGTA
M29MutLZ	CATCAGCTCGCTAACCAGCAGGCTGACAACGCTC GTCGCAAGGAGCACATCGCTGAAGACCAGAACAG CTTCGCTTGCCGCATG	CATGCGGCAAGCGAAGCTGTTCTGGTCTTCA GCGATGTGCTCCTTGCGACGAGCGTTGTCAG CCTGCTGGTTAGCGAGCTGATG
M29Mut NLS	ACATTCTCGGCGGCG CGGGGAGGG	CCCTCCCCGCGCCGCCGAGAATGT
OsMADS27cds	CACCATGGGGAGGGGGAAGATTGTGATCC	TGGATTCAACTGTAACCCTAGCTTA
OsMADS14cds	CACCATGGGGCGGGGCAAGGTGCAGCTG	CATAACAATGGAAAATTGCCAT
OsMADS15cds	CACCATGGGAGAGGATCT AGAATCCCTG	AGCATTGAGGTGGCTCAG CATCCAT
OsMADS16cds	CACCATGGGGAGGGGCAAGATCGAGATC	ACCGAGGCGCAGGTCGTGGTTGCCG
OsMADS17cds	CACCATGGATCGATCAGA GATGGGGAGG	GAGGGGCCATCCCATCACAAAGTT
OsMADS18/28cds	CACCATGGGGAGAGGGCCGGTGCAGCTGC	TGTGTGACTTGTCCGGAG CATCCAT
OsMADS1cds	CACCATGGGGAGGGGGAAGGTGGAGCTG	TATCCAGCCGGATGGGAT GTGTT
OsMADS22cds	CACCATGGCGCGGGAGAGGCGGGAGATA	CTTCCATGCACC ACA AGG CAG CCC
OsMADS25cds	CACCATGGGGAGAGGGAAGATTGCCATC	TTCATCTTCAACTTCTTTTTGACTC
OsMADS26cds	CACCATGGCGCGAGGCAAGGTGCAGCTC	GAAGGAATAGCCCATCTC CGAG
OsMADS30cds	CACCATGGGGCAAGGGAAGATCGAGATGA	TATAAACCAGAATCGCCA AG
OsMADS31cds	CACCATGGGGCGTGGGAGAGTAGAGCTCA	AAACTTGATTATTTCAATATAGTGT
OsMADS32cds	CACCATGGGGAGGGGGCGCAGCGAGATAA	GTTGAACCCCAGTTTAAGGTCCAAG
OsMADS33cds	CACCATGGTCAGAGGAAA GGTGCAAATGC	TGAGAAGAAGCGATTTCC GTAGGTC
OsMADS34cds	CACCATGGGGCGAGGCAAGGTGGTGCTT	TTCCTGATAGTTGTTCTGCATCTCA
OsMADS37cds	CACCATGGAGGGAGGAGGGAGGAGGAGG	AGAGCTCACTCCTGATCT TGGCTC
OsMADS3cds	CACCATGATGAACATGATGACCGATCTG	GGCAAGCCGAGTTAGACT GTTTG
OsMADS4cds	CACCATGGGGCGCGGCAAGATCGAGAT	CTTCTCCTGCTGGAGGTT GGGGT
OsMADS50cds	CACCATGGTGCGGGGGAAGACGCAGATGAA	AGAATGGGGCATCGCTTG GCTATCT
OsMADS55cds	CACCATGGCGAGGGAGAGGAGGGAGATA	AGGCAACCCTAGTTTCAG GGATAT
OsMADS56cds	CACCATGGTGCGGGGGAGGACGGAGCTGA	ACCTGTCTCCGACCGGTT GGAGGA
OsMADS5cds	CACCATGGGGCGAGGGAAAGTAGAGCTGA	TTGGTTGAGGTGATCCAT GTAAGC
OsMADS60cds	CACCATGGAAAAGGCCGCGGCCAATCAGG	TATCTCTCTGTAAAGGTTGAGCCTG
OsMADS65cds	CACCATGGCGCGGAGGGGGAGAGTGCAG	TGCACTTCCTTCCTCCTG CCCCCTA
OsMADS66cds	CACCATGCACATATACAA AGAGCAGGAGGC	GTATACCTGGGCATTGAT CTCTGCA
OsMADS6cds	CACCATGGGGAGGGGAAGAGTTGA	AAGAACCCATCCCAGCAT GAAGTT
OsMADS7/45cds	CACCATGGGGAGGGGTCGGGTGGAGCTGA	TGGTAGCCATGGGGGCATGTAGGTGTT
OsMADS8/24cds	CACCATGGGGAGAGGGAGGGTGGAGCTG	GGGTAGCCATGTCGGCAT GAAGGCA
OsMADS13cds	CACCATGGGGAGGGGCAGGATTGAGA	GAAGTGAGGAGGCGGCGCGTCGG
OsRR14cds	CACCATGGCTCGCAAGATGATC ATGGTG	AGCAAGTCTAAATTTGGC AAGGATGACTC
OsMADS21cds	CACCATGGGGAGGGGGAAGATTGAGATAAAGAGA	GCCTTTGCCGGAGTCGAC CTTGAA
OsMADS29cds	CACCATGGGGCGCGGCAAGAT	CCACAGCTGCAGGCCGTG
